# Conduction defects and arrhythmias in *mdx* mice are not associated with a degeneration of the cardiac Purkinje network

**DOI:** 10.3389/fphys.2025.1607916

**Published:** 2025-06-26

**Authors:** Juliette Vahdat, Jakob Sauer, Jessica Marksteiner, Karlheinz Hilber, Lucile Miquerol

**Affiliations:** ^1^ Aix-Marseille Université, CNRS UMR 7288, Developmental Biology Institute of Marseille, Marseille, France; ^2^ Department of Neurophysiology and Neuropharmacology, Center for Physiology and Pharmacology, Medical University of Vienna, Vienna, Austria

**Keywords:** conduction system anatomy, DMD, Purkinje fibers, ECG, sodium current

## Abstract

Duchenne muscular dystrophy (DMD) is a severe X-chromosomal disease characterised by progressive muscle weakness and degeneration. Cardiac involvement is inevitable in DMD patients and ventricular arrhythmias are a high-risk factor for mortality in these patients. Ventricular arrhythmias are often triggered by a dysfunctional ventricular conduction system, which serves as an electrical circuit in the heart to ensure the synchronization of the heartbeat. This system includes Purkinje fibers which are susceptible to degeneration in DMD patients, leading to cardiac conduction disorders. To unravel whether a defective ventricular conduction system may account for arrhythmogenesis in a DMD mouse model, we performed a longitudinal study of the cardiac electrical activity in *mdx* mice. ECG recordings showed a progressive increase in PR interval over time and a prolonged QRS in *mdx* compared to wild-type (WT) mice. At baseline, only *mdx* mice presented premature ventricular complexes (PVC), and a greater prevalence of PVC was observed after β-adrenergic stimulation in these mice. These conduction defects and arrhythmias occurred while no defects in the morphology and maturation of the Purkinje fiber network were observed. However, *mdx* mice had a larger heart and showed signs of fibrosis and hypertrophy. Furthermore, conduction defects in *mdx* mice were associated with ventricular dyssynchrony and sodium current (I_Na_) reduction in ventricular myocytes and Purkinje fibers. Altogether, these data demonstrated that *mdx* mice develop a progressive arrhythmogenic cardiomyopathy in association with I_Na_ loss, ventricular fibrosis but without degeneration of the ventricular conduction system.

## Introduction

Duchenne muscular dystrophy (DMD) is primarily characterized by skeletal muscle degeneration resulting from mutations in the X-linked gene encoding the structural cytoskeletal Dystrophin. Besides this skeletal muscle degeneration, cardiomyopathy is highly prevalent in DMD patients, being observed in 50% of patients by the age of 10 and almost 100% by adulthood (Nigro et al., 1990). DMD-associated cardiomyopathies are prone to ventricular arrhythmias and lead to chronic congestive heart failure, which is now the leading cause of death in DMD patients (Kamdar and Garry, 2016; Shirokova and Niggli, 2013; Spurney, 2011; Tsuda and Fitzgerald, 2017). As the specific mechanisms behind these arrhythmias are poorly understood, current treatment strategies do not prevent life-threatening ventricular tachycardias.

Various cardiac arrhythmias, recorded by ECG in DMD patients, implicate dysfunction of the ventricular conduction system (VCS) (Chenard et al., 1993; Perloff, 1984). The VCS is composed of the His bundle, bundle branches (BB) and ends in a complex network of Purkinje fibers (PF) and is responsible for synchronizing the heartbeat. Left bundle branch block (LBBB) is relatively frequent in DMD patients and is a major predictive factor for cardiac events and mortality (Fayssoil et al., 2018). Moreover, dystrophin-deficient Purkinje fibers have been reported to exhibit vacuole degeneration in patients with DMD (Nomura and Hizawa, 1982) and in dystrophic dogs (Urasawa et al., 2008; Echigoya et al., 2017). In addition, dystrophin protein rescue in cardiac Purkinje fibers contributed to the improvement or prevention of conduction abnormalities in the dystrophic dog heart (Echigoya et al., 2017). Thus, dystrophin may play a primary role in the function and integrity of cardiac Purkinje fibers. Besides, dystrophin is more abundantly expressed in human Purkinje fibers in comparison with contractile cardiomyocytes (Bies et al., 1992). Among the multiple causes of conduction defects, we have previously shown that morphological defects of the Purkinje network induce a slow conduction and ventricular dyssynchrony in mice, leading to ventricular dysfunction (Choquet et al., 2018; Choquet et al., 2023; Meysen et al., 2007). However, the structure of the ventricular PF network in *mdx* mice, the most commonly used animal model for DMD (Bulfield et al., 1984), is unknown.

Dystrophin-deficient (*mdx*) mice share important clinical features with the cardiomyopathy of DMD patients (Chu et al., 2002). Electrical disturbances such as reduced PR, PQ and elongated QRS have been observed in *mdx* males and females while dilated cardiomyopathy with cardiac dysfunction arises with age (Branco et al., 2007; Koenig et al., 2014). In *mdx* mice, conduction defects have been assigned to multiple causes such as abnormal calcium homeostasis, elevated reactive oxygen species or impaired sodium current (I_Na_) in cardiomyocytes (Gavillet et al., 2006; Koenig et al., 2011; Wang et al., 2018). Our previous studies have indeed shown that both mdx ventricular cardiomyocytes from the working myocardium and mdx Purkinje fibers have abnormally diminished I_Na_ densities (Ebner et al., 2022; Ebner et al., 2020; Koenig et al., 2011; Sauer et al., 2024). However, this does not explain all conduction defects observed in DMD patients. A core issue for the cardiac pathogenesis in DMD is to determine whether defective morphogenesis of the conduction system may explain the life-threatening arrhythmogenesis and associated heart failure of these patients. To answer this question, we studied cardiac function and the morphology of the PF network in *mdx* mice in parallel by crossing them with Cx40-GFP mouse line in which GFP is specifically expressed in the entire ventricular conduction system (Miquerol et al., 2004).

## Materials and methods

### Ethics statement

All studies and procedures involving animals were in strict accordance with the recommendations of the European Community Directive Article (2010/63/UE) for the protection of vertebrate animals used for experimental and other scientific purposes. The project was specifically approved by the regional ethics committee and by the French Ministry of Research (APAFIS N° 36487-2022040816108385 v.7). All experimental protocols for the patch clamp studies were approved by the Austrian Science Ministry (BMWFW-66.009/0175-WF/V/3b/2015).

### Mouse models

Dystrophin-deficient *mdx* mice on the BL10 background (C57BL/10ScSn-Dmdmdx/J) (Ebner et al., 2020) were cross-bred with a transgenic mouse line (Cx40eGFP/+; BL10 background) expressing eGFP under the control of the connexin 40 (Cx40) gene (Miquerol et al., 2004). Only males aged between 1 and 12 months were used for the experiments. Genotyping of the mice was performed using standard PCR assays.

### Macroscopic and histological analyses

Mice were euthanized by cervical dislocation and hearts from 12-month-old animals were excised and immediately received a perfusion of PBS-KCl (50 mM) by the aorta.

For histological studies, adult hearts were fixed overnight in 4% paraformaldehyde (vol/vol) in PBS, washed in sucrose gradient, then embedded in OCT and cryosectioned. For immunofluorescence, sections were permeabilized in PBS 1X/0.2% Triton X100 for 20 min and incubated for 1 h in saturation buffer (PBS 1X/3% BSA/0.1% Triton X100). Primary antibodies were incubated in saturation buffer overnight at 4°C. Secondary antibodies coupled to fluorescent molecules were incubated in saturation buffer and after washes, hearts were observed under a Zeiss Apotome microscope.

For whole-mount immunofluorescence, the left ventricular wall of adult hearts was opened by scissors and pinned on a petri dish to expose the septal surface and fixed in 4% paraformaldehyde for 2 h at 4°C, washed in PBS, permeabilized in PBS 1X/0.5% Triton X100 for 1 h and incubated for 3 h in saturation buffer (PBS 1X/3% BSA/0.1% Triton X100). The primary antibodies were incubated in saturation buffer for 24 h at 4°C. Secondary antibodies coupled to fluorescent molecules were incubated in saturation buffer and after washes, hearts were observed under a Zeiss LSM780 confocal microscope.

Antibodies used in this study were specific to Contactin-2 (AF1714, R&D system), GFP (AbD Serotec), dystrophin (12715-1-AP, ProteinTech), WGA-Cy3 (29076-1, Clinisciences) and WGA-Cy5 (29024-1, Clinisciences). The antibody against Cx43 is homemade and previously described (Gros and Jongsma, 1996).

### Fibrosis measurements

Wheat Germ Agglutinin (WGA) staining was quantified using Fiji (Fiji Is Just ImageJ). Images were converted to 8-bit grayscale, and a manual threshold was applied to isolate the WGA-positive signal. Threshold values were adjusted consistently across all samples, based on intensity histograms and visual observation, to ensure that the stained regions were accurately defined. A region of interest (ROI) was manually delineated to include only cardiac tissue, excluding background and non-specific areas. Within the ROI, the number of WGA-positive pixels was expressed as a percentage of the total number of tissue pixels: (number of WGA-positive pixels/total number of tissue pixels) × 100.

### Surface electrocardiography

Surface ECGs were performed on anesthetized mice. An induction with 5% isoflurane was followed by maintenance at 1%–2% in a constant flow of oxygen at 700 mL/min. ECGs were recorded with a bipolar system in which the electrodes were placed subcutaneously at the right (negative) and left forelimb (reference) and the left hindlimb (positive) for lead II, at the right (reference) and left forelimb (negative) for lead III. Electrodes were connected to a Bioamp amplifier (AD Instruments) and were digitalized through a PowerLab 26T (AD Instruments). Digital recordings were analyzed with LabChart software version 8.1.13 (AD Instruments). Events were registered to 100 K/s and were filtered to 50 Hz. ECG recordings were obtained for 3 min after stabilization of the signal. Post-analysis was performed for heart rate, PR, QRS, QT intervals, T, R and S durations and T, R, S and QRS amplitudes. Body temperature was monitored using a temperature probe and maintained above 36°C and a warm pad (A-2101-00298, Intellibio). The two first electrocardiograms were recorded at 1 month and 3 months of age, then every 3 months until 12 months of age.

An Isoproterenol (ISO) stress test was performed at 12 months of age. After recording an ECG as described above, mice received a single intraperitoneal injection of ISO at a dose of 2 μg/g body weight. ISO was prepared as follows: DL-Isoproterenol hydrochloride (I5627, Sigma) was dissolved in ddH2O and vortexed to provide a 10 μg/μL ISO stock solution.

Vector cardiograms were obtained based on lead I (X-axis) and aVF (Y-axis) (lower) and represent the orientation of the main electrical axis of the heart.

### Cardiomyocyte isolation

Cardiac Purkinje fibers were isolated from wild-type (WT)- and *mdx*-*Cx40* ^eGFP/+^ mice as previously described (Ebner et al., 2020). The mice were anesthetized with isoflurane (2%, inhalation) and killed by cervical dislocation. After excision of the heart, a cannula was inserted into the aorta. The heart was then retrogradely perfused with calcium-free solution comprising 0.17 mg/mL Liberase TH (Roche) at 37°C for 18 min using a Langendorff setup. To further liberate Purkinje fibers, a second digestion step was applied, for which the ventricles were cut open along the aorta and placed in a culture dish containing 0.17 mg/mL Liberase TH (in calcium-free solution) for 8 min at room temperature. The tissue was then pulled into small pieces and incubated on a shaker at 37°C. Over 30 min, the Ca concentration was increased to 150 μM in four steps. The cells were then resuspended in Minimum Essential Medium (MEM)-α containing ITS media supplement (diluted 1:100), 2 mM L-glutamine, 100 U/mL penicillin, 0.1 mg/mL streptomycin and 17 μM blebbistatin (Sigma-Aldrich). Cells were then seeded on Matrigel (Corning)-coated 3.5 cm culture dishes for electrophysiological recordings. For experiments with ventricular cardiomyocytes of the working myocardium, hearts from mdx mice lacking Cx40-controlled eGFP expression were used for cell isolation. The procedure was as for Purkinje fiber isolation, except for the use of only a single digestion step, for which the hearts were perfused with 0.17 mg/mL Liberase TH (in calcium-free solution) at 37 °C for 10 min using a Langendorff setup (Hugo Sachs Elektronik).

### Sodium current recordings

I_Na_ in isolated cardiac Purkinje fibers and ventricular cardiomyocytes of the working myocardium was recorded up to 6 h after cell isolation utilizing the whole cell patch clamp technique. The measurements were performed at room temperature (22°C ± 1.5 °C) using an Axopatch 200B patch clamp amplifier, a Digidata1440 digitizer and Clampex 10.7 software (Axon Instruments, Union City, CA, United States). Patch pipettes were formed with a P-97 horizontal puller (Sutter Instruments, Novato, CA, United States) from aluminosilicate glass capillaries (A120-77-10; Science Products, Hofheim, Germany). They were filled with a solution consisting of (in mM) 5 NaCl, 110 CsF, 10 EGTA and 10 HEPES, adjusted to pH 7.3 with CsOH. Tip resistances lay between 1 and 1.5 MΩ. The bath solution contained (in mM) 5 NaCl, 135 N-methyl-D-glucamine, 2.5 KCl, 1 CaCl_2_, 1 MgCl_2_, 10 HEPES and 0.017 blebbistatin, adjusted to pH 7.4 with HCl. Fresh bath solution was continuously administered to the patched cells using a DAD-8-VC superfusion system (ALA Scientific Instruments, Westbury, NY, United States). Purkinje fibers were identified by their eGFP signal and morphological characteristics, as previously described (Ebner et al., 2020). I_Na_ was activated by 25 ms depolarizations ranging from −87 to −7 mV. Voltages were corrected for the liquid junction potential. Recordings were low-pass filtered with 10 kHz and digitized at 35 kHz. Analysis of the data was carried out with Clampfit 10.7 (Axon Instruments) and GraphPad Prism 8 (San Diego, CA, United States). I_Na_ peaks were measured and divided by the membrane capacitance to calculate current densities. To obtain current density-voltage relationships, these values were then plotted against the test pulse voltages. For curve fitting, the following function was used: I = G_max_·(V-V_rev_)/(1+exp ((V_50_-V)/K)), where I is the current, G_max_ is the maximal conductance, V is the membrane potential, V_rev_ is the reversal potential, V_50_ is the voltage at which the half-maximal activation occurred, and K is the slope factor.

### Statistical analysis

Data are expressed as means ± standard deviation (SD). The I_Na_ data are expressed as means ± standard error (SE). Significant differences for electrocardiogram parameters were determined using two-way analysis of variance (ANOVA) followed by Sidak *post hoc* testing. Significant differences for heart size were determined using an unpaired t-test. All tests were made with Graphpad Prism software (Graphpad Prism 9.5.1, La Jolla, CA, United States). A nested analysis respecting the hierarchical data structure (Sikkel et al., 2017) was used for statistical comparisons of the patch clamp data. A p-value <0.05 was considered statistically significant.

## Results

### Conduction defects and arrhythmias in mdx mice increase with age

**FIGURE 1:**
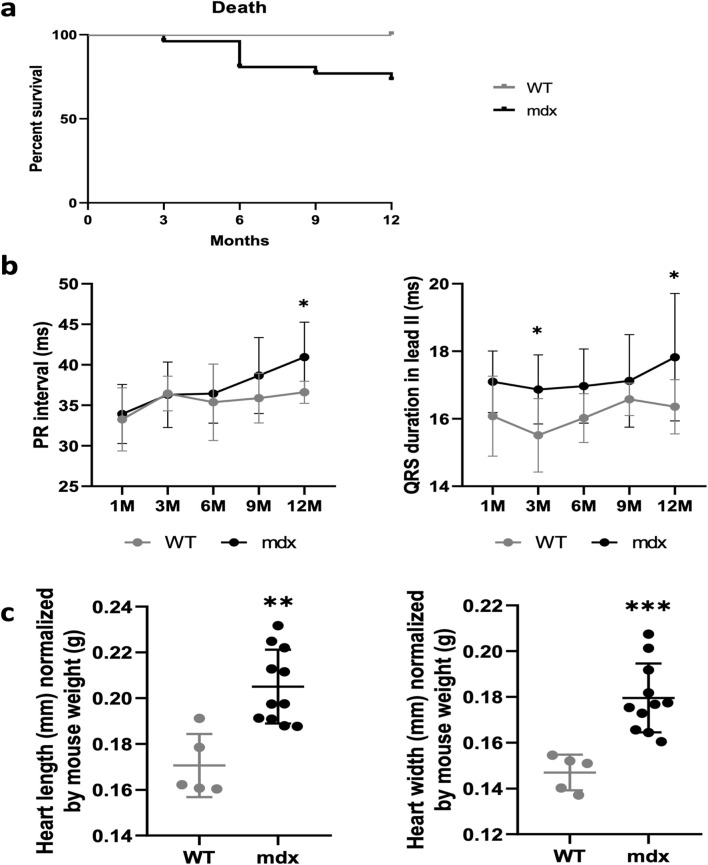
Progressive cardiac conduction defects in *mdx* mice. **(a)** Kaplan-Meier survival plot. The survival curves showed that 27% of the *mdx* mice (n = 26) died at 12-month-old, whereas all WT survived (n = 9). **(b)** Measurements of cardiac parameters in WT and *mdx* mice by electrocardiography. Graphs representing the evolution of the PR-II and QRS-II intervals measured in the same mice over a year show a progressive increase in these parameters in *mdx* (n = 26) compared to WT (n = 9) mice. **(c)** Graphs representing heart size measurements in WT (n = 5) and *mdx* mice (n = 11). *Mdx* mice present a significantly greater heart length and width compared to WT. *p < 0.1; **p < 0.01; ***p < 0.001.

In order to evaluate conduction defects in mdx mice we crossed mice carrying the mdx mutation with a Cx40-GFP allele to generate double transgenic mice mdx::Cx40-GFP (C57BL/10ScSn-Dmdmdx/J). Cx40-GFP mice express the GFP reporter gene under the control of the gap junction alpha 5 (Gja5) promoter (Miquerol et al., 2004). Gja5 encodes for Connexin 40 specifically expressed in atrial cardiomyocytes and in the ventricular conduction system (VCS). Firstly we evaluated the survival rate of these mice to study the age-related progression of DMD associated cardiac defects. We observed that 27% of *mdx::Cx40-GFP* mice died after 1 year while 100% of control animals survived (Figure 1a). A follow-up of the cardiac activity was made through six-lead surface electrocardiogram (ECG) recordings on mice anesthetized with isoflurane. The two first electrocardiograms were recorded at 1 month and 3 months of age, then every 3 months until 12 months of age (Figure 1b). The *mdx::Cx40-GFP* mice presented a statistically significant increased PR interval compared to controls at 12-month-old, indicative of first degree atrioventricular block (36.6 ± 1.4 ms for WT vs. 41.5 ± 4.6 ms for mdx). QRS-II duration in *mdx::Cx40-GFP* mice was significantly increased compared to the controls at 3-month (16.1 ± 1.2 ms for WT vs. 17.1 ± 0.9 ms for *mdx*) and at 12-month (16.3 ± 0.8 ms for WT vs. 17.9 ± 1.8 ms for *mdx*), indicating a slower ventricular activation in the *mdx::Cx40-GFP* mice. The other ECG parameters did not present any differences between *mdx* and WT mice (Table 1). As expected, *mdx::Cx40-GFP* mice presented a dystrophic myocardial phenotype. Normalization of the heart size by the body weight of each mouse revealed a greater heart length (0.17 ± 0.01 for WT vs. 0.20 ± 0.02 for mdx) and a greater heart width (0.15 ± 0.01 for WT vs. 0.18 ± 0.02 for mdx) in *mdx::Cx40-GFP* mice (Figure 1c).

**TABLE 1:** Surface ECG parameters in Lead II.

Age	1-month-old		3-month-old		6-month-old		9-month-old		12-month-old	
Genotype	WT	mdx	WT	mdx	WT	mdx	WT	mdx	WT	mdx
N	9	26	9	26	9	25	9	21	9	20
RR (ms)	146 ± 16.9	141.3 ± 15.6	153.3 ± 15.4	138.8 ± 27.2	146 ± 13.7	141.4 ± 19.5	137.5 ± 16.6	128.7 ± 18.3	141.6 ± 13.9	133.4 ± 19.0
PR (ms)	33.2 ± 3.9	33.9 ± 3.6	36.5 ± 2.1	36.3 ± 4.0	35.4 ± 4.7	36.4 ± 3.6	35.9 ± 3.1	38.7 ± 4.7	36.6 ± 1.4	41.6 ± 4.6*
P (ms)	15.9 ± 1.1	16.7 ± 1.4	17.1 ± 1.3	18.2 ± 2.5	15.8 ± 1.4	17.3 ± 1.6	15.4 ± 2.6	17.1 ± 1.9	16.4 ± 1.4	17.8 ± 1.8
QRS-I (ms)	15.2 ± 0.9	16.3 ± 1.4	15.1 ± 0.7	15.7 ± 1.2	16 ± 1.9	16.3 ± 0.8	15.7 ± 0.9	17.1 ± 1.6	15.8 ± 1.1	17.7 ± 1.9**
QRS-II (ms)	16.1 ± 1.2	17.1 ± 0.9	15.5 ± 1.1	16.9 ± 1.0*	16 ± 0.7	17 ± 1.1	16.6 ± 0.5	17.1 ± 1.4	16.3 ± 0.8	17.9 ± 1.8*
QRS-III (ms)	18.2 ± 2.1	17.5 ± 2.3	16 ± 1.9	17.5 ± 1.5	15.7 ± 1.6	17.3 ± 1.1	16 ± 1.5	17.4 ± 1.3	16.4 ± 2.0	18.0 ± 1.4
QT (ms)	39.7 ± 3.0	39.3 ± 2.7	40.8 ± 5.2	41.5 ± 4.5	40.6 ± 4.2	42.7 ± 3.9	41 ± 3.5	40.3 ± 3.8	45.6 ± 3.3	43.4 ± 4.1
P (µV)	65.4 ± 22.2	63.3 ± 19.2	53.8 ± 14.5	49.7 ± 13.8	44.7 ± 11.6	41.8 ± 11.3	44.6 ± 30.7	39.3 ± 11.4	41.9 ± 11.8	38.8 ± 14.6
R (µV)	692.9 ± 129.9	641.2 ± 117.4	585.4 ± 134.0	521.7 ± 102.3	505.9 ± 106.0	447.7 ± 90.3	509.7 ± 131.0	403.3 ± 99.6	494.4 ± 92.7	392.6 ± 111.5
S (µV)	−133.5 ± 43.5	−188.3 ± 76.0	−105.3 ± 54.2	−119.8 ± 89.3	−98.9 ± 49.8	−75.8 ± 71.4	−75.5 ± 38.6	−64.2 ± 62.9	−83.8 ± 62.0	−55.4 ± 65.3

*p < 0.05; **p < 0.01.

**FIGURE 2:**
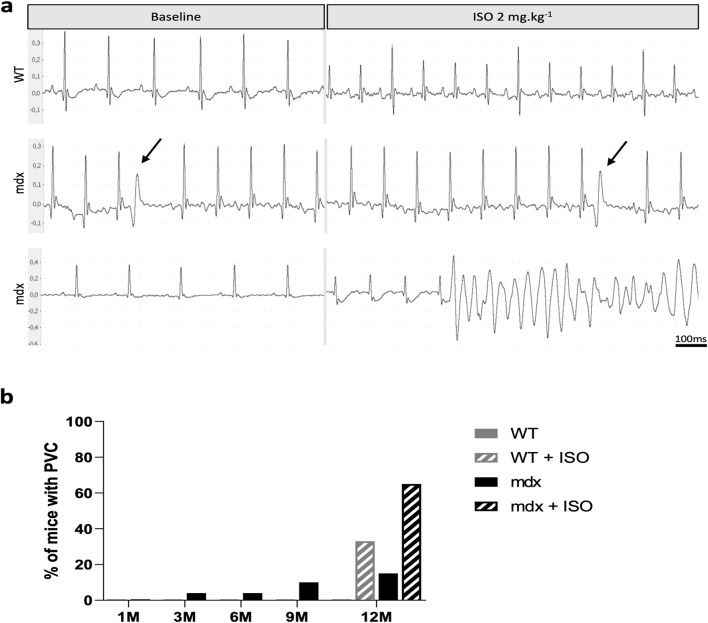
Spontaneous ventricular arrhythmic events in *mdx* mice. **(a)** Representative tracings from surface ECG measured in lead II in anaesthetized mice before and after ISO (2 mg/kg) IP injection. At baseline, only *mdx* mice present premature ventricular complexes (PVC, black arrow). After ISO challenge, the cardiac rhythm is accelerated in all mice and PVCs and ventricular tachycardia are mainly observed in *mdx* mice (VT). **(b)** Histograms showing that PVCs are more frequent in *mdx* mice with age. ISO stimulation revealed a greater prevalence of PVC in *mdx* mice compared to WT at 12 months of age. WT (n = 9) and *mdx* mice (n = 26).

During ECG follow-up, we scored spontaneous ventricular arrhythmic events and found that *mdx::Cx40-GFP* but not WT mice presented premature ventricular complexes (PVC) under basal conditions (Figures 2a,b). To mimic physiological stress, the mice were subjected to β-adrenergic stimulation by injection of Isoproterenol (2 μg/g) at the age of 12 months. The increase in heart rate after Isoproterenol injection was not significantly different between WT and *mdx::Cx40-GFP* mice (28.8% ± 9.0% of increase for WT vs. 26.66% ± 10.3% of increase for *mdx*). β-adrenergic stimulation increased the number of mice with PVC in both groups, with a greater prevalence in *mdx::Cx40-GFP* mice (33% for WT vs. 65% for mdx) (Figure 2b). One *mdx::Cx40-GFP* mouse displayed ventricular tachycardia (VT) (Figure 2a). In summary, the progressive onset of cardiomyopathy in *mdx::Cx40-GFP* mice is associated with a larger heart, ventricular conduction defects and arrhythmias at 12 months of age.

### Preserved Purkinje fiber network in mdx::Cx40-GFP mice

**FIGURE 3:**
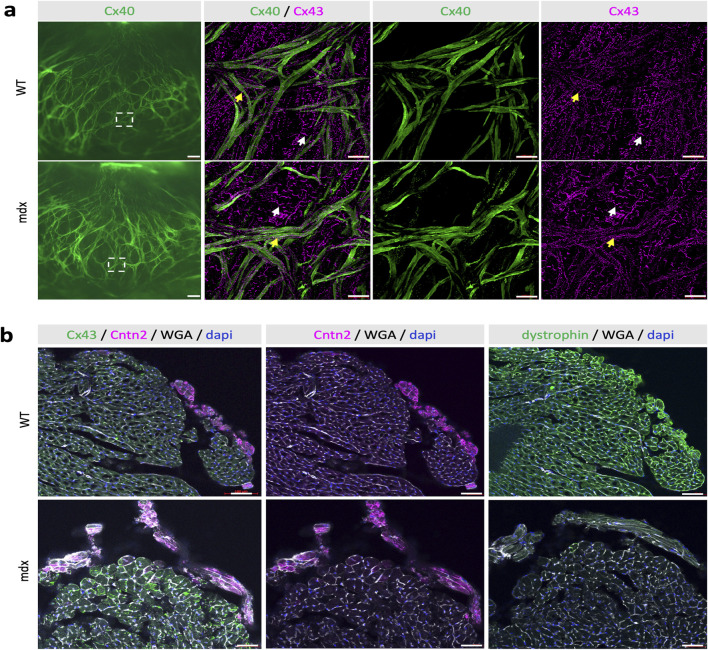
No morphological and maturation defects of the Purkinje Fiber network enriched in dystrophin. **(a)** Whole-mount immunofluorescence with Cx43 antibodies and Cx40-GFP on opened LV from WT and *mdx* adult mice. On the left image, the GFP fluorescence indicates a similar pattern of the Purkinje Fiber network between *mdx* and WT mice. Scale bar = 500 µm. Higher magnifications indicated by squares show the normal expression of the gap junction Cx43 at the intercalated discs (IDs) in contractile cardiomyocytes (white arrows) or along the membrane in PF (Yellow arrows) in both *mdx* and WT mice. Scale bar = 100 µm (WT n = 3; mdx n = 9). **(b)** Immunofluorescence with Contactin-2, Cx43, DMD and WGA-cy3 antibodies on transversal sections at the mid-ventricular level from WT and *mdx* mice. While Cntn2 expression, the marker of VCS maturation is similar in WT and *mdx* mice, DMD is absent in mdx hearts and enriched in their Purkinje fibers compared to other cardiomyocytes in WT. WGA and Cx43 staining at the membrane highlight the cardiac hypertrophy seen in *mdx* hearts. Scale bar = 50 µm (n = 11).

Since Purkinje fiber degeneration has been observed in human and canine DMD (Urasawa et al., 2008; Echigoya et al., 2017; Nomura and Hizawa, 1982), we used the *Cx40-GFP* mouse line to study the morphology and histology of Purkinje fibers in *mdx::Cx40-GFP* mice. In these mice GFP is expressed in the entire VCS including the His bundle, bundle branches and the Purkinje fiber network as seen in a luminal view of the endocardial surface of an opened WT left ventricle (Figure 3a). The PF network in *mdx::Cx40-GFP* mice is similar to WT mice in terms of number, distribution and structure of ellipsoids. Thus, there was no difference in the morphology of the Purkinje fiber network between control and *mdx::Cx40-GFP* mice (Figure 3a). As recent data have suggested that Cx43 lateralization contributes to DMD arrhythmogenesis in *mdx* mice (Gonzalez et al., 2015), we performed whole-mount immunostaining with a Cx43 antibody to examine the distribution of Cx43 gap junctions in working cardiomyocytes and in Purkinje fibers. Under high magnification, Cx43 gap junctions are present mainly at the intercalated discs (ID) of working cardiomyocytes, whereas they are distributed all along the plasma membrane of Purkinje fibers in WT (Figure 3a). We found a similar distribution of Cx43 gap junction localisation in *mdx::Cx40-GFP* hearts in both PF or working myocardium (Figure 3a). These data show that the Purkinje fiber network structure and the overall distribution of Cx43 are not affected in *mdx::Cx40-GFP* mice at 12 months of age when the arrhythmia-associated cardiomyopathy is in place.

To further investigate this, we performed histological analysis of Purkinje fibers on cryosections of WT and *mdx* hearts. Purkinje fibers were identified using a contactin-2 (Cntn2) antibody and cardiomyocytes were identified using DMD and WGA (Wheat germ agglutinin) staining. In WT hearts, DMD is present on the membrane of all cardiomyocytes with higher levels found in Purkinje fibers (Figure 3b). As expected, no staining with DMD antibody was detected in *mdx* hearts (Figure 3b). WGA staining showed that the membrane and the size of cardiomyocytes are overall larger in *mdx* hearts compared to WT. Cntn2 is a well-known marker of mature Purkinje fibers and play an important role in the electrical propagation in the heart (Pallante et al., 2010). Cntn2 staining was similar in WT and *mdx* hearts (Figure 3b), demonstrating the preservation of mature Purkinje fibers in the murine DMD model.

### Ventricular conduction defects in mdx mice are associated with I_Na_ reduction in ventricular cardiomyocytes and Purkinje fibers over a wide animal age range

Slowed ventricular conduction, as represented by QRS interval prolongation in the ECGs of *mdx* mice, may result from reduced I_Na_ in *mdx* compared to WT in ventricular cardiomyocytes and Purkinje fibers. Here, we addressed whether the age of mice (range: neonatal – 1-year-old) has an impact on the severity of I_Na_ loss in the dystrophic *mdx* mouse heart. In Koenig et al. (2011), we reported that ventricular cardiomyocytes isolated from neonatal *mdx* mice show less severe I_Na_ loss than ventricular myocytes from adult (4-6-month-old) *mdx* mice. This suggested that, although already present at neonatal age, I_Na_ loss in dystrophic ventricular cardiomyocytes becomes more pronounced at adulthood. This result was in line with a normal QRS interval in neonatal, but prolonged QRS interval in adult *mdx* compared to WT mice (Koenig et al., 2011).

**FIGURE 4:**
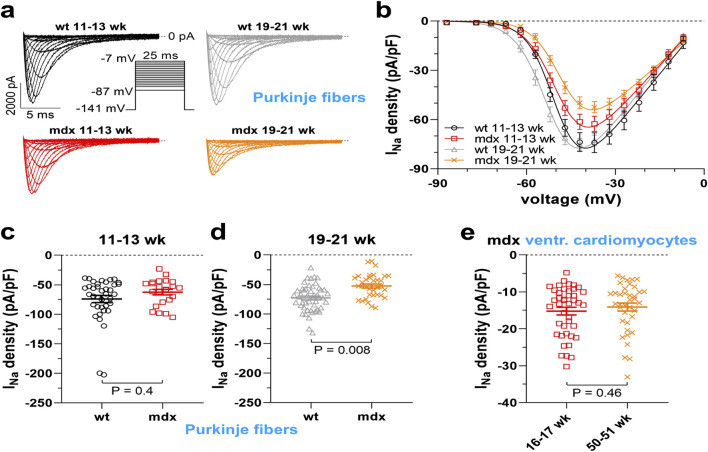
Sodium current (I_Na_) densities in cardiac Purkinje fibers and ventricular cardiomyocytes from dystrophin-deficient mdx and wild-type (WT) mice at different animal ages. **(a)** Representative original whole cell I_Na_ traces recorded from Purkinje fibers from 11–13- or 19–21-week-old WT and *mdx* mice. The pulse protocol to elicit the currents is shown in the inset. **(b)** Current density-voltage relationships derived from a series of experiments as displayed in a (38 cells, six animals, WT 11–13 weeks; 23 cells, five animals, mdx 11–13 weeks; 48 cells, six animals, WT 19–21 weeks; 34 cells, five animals, mdx 19–21 weeks). The solid lines represent fits with a function given in the methods section. **(c)** Statistical comparison of current density values at −37 mV between WT and mdx Purkinje fibers from 11–13-week-old mice. In this age range, current densities in mdx Purkinje fibers (−62.6 ± 4.7 pA/pF) were only reduced by trend when compared to WT Purkinje fibers (−74.1 ± 6 pA/pF). **(d)** Comparison of current density values at −37 mV between WT and mdx Purkinje fibers from 19–21-week-old mice. Here, a significant difference between WT (−72.7 ± 3.3 pA/pF) and mdx (−52.5 ± 3.5 pA/pF) Purkinje fibers existed. **(e)** I_Na_ densities at −37 mV of ventricular cardiomyocytes of the working myocardium isolated from 16–17-week-old (42 cells from three animals) or 50–51-week-old (38 cells from three animals) mdx mice. There was no significant difference (−15.2 ± 1 pA/pF vs. −14.1 ± 1.1 pA/pF, 16–17 weeks vs. 50–51 weeks). Values represent means ± SE. A nested analysis respecting the hierarchical data structure was used for statistical comparisons (Sikkel et al., 2017).

Here, in Figures 4a–d, the I_Na_ properties of Purkinje fibers derived from 11–13-week-old and 19–21-week-old WT and *mdx* mice were compared. We observed that the current densities of Purkinje fibers in WT mice were independent of the age of the animal. At 11–13 weeks, current densities in *mdx* Purkinje fibers were only slightly reduced compared to those in WT Purkinje fibers (Figure 4c). In contrast, at 19–21 weeks, *mdx* Purkinje fibers showed a significantly reduced current density (Figure 4d). This suggests an increase in I_Na_ loss in Purkinje fibers with age in dystrophic *mdx* mice.

Finally, we tested whether the loss of I_Na_ was even more severe in old *mdx* mice, an age known to be associated with the onset of arrhythmogenic cardiomyopathy (Quinlan et al., 2004). In Figure 4e the I_Na_ densities of ventricular cardiomyocytes derived from 16–17 and 50–51-week-old *mdx* mice were similar.

Taken together, our I_Na_ recordings suggest that current loss in dystrophic ventricular cardiomyocytes and Purkinje fibers is already present in *mdx* mice at a very young age, worsens from juvenile to full adulthood, and finally persists with similar severity until 1 year of age, when arrhythmogenic cardiomyopathy is present.

### Conduction defects are associated with ventricular dyssynchrony and fibrosis in mdx::Cx40-GFP mice

**FIGURE 5:**
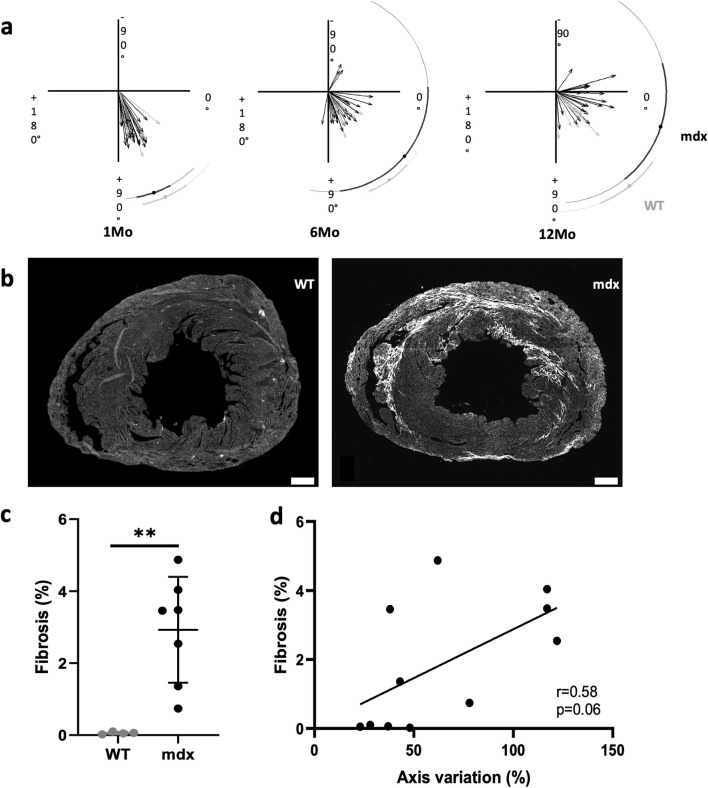
Arrhythmogenic cardiomyopathy associated with ventricular dyssynchrony and fibrosis. **(a)** The cardiac electrical axis calculated from surface electrocardiograms is normally comprised between 0° and 120° in WT while a progressive deviation towards the left is observed with age in *mdx* mice (WT n = 9; *mdx* n = 26). **(b)** WGA staining by immunofluorescence shows the presence of cardiac fibrosis only in *mdx* mice. Scale bar = 500 µm. **(c)** Fibrosis was quantified by measuring the percentage of WGA positive area in the LV and is significantly increased in *mdx* (n = 7) mice in comparison to WT (n = 4)-II (unpaired t-test, **p = 0.0042). **(d)** Cardiac fibrosis correlates with cardiac axis deviation in *mdx* mice (n = 11) (r = 0.58; p = 0.06).

To detect any other cardiac activation defects, we calculated the angle of the main electrical axis of the heart from ECG recordings for each mouse. At 1 month of age, all WT and *mdx* mice presented a normal axis comprised between 0° and 120° (Figure 5a). At 6 months of age, three out of the 26 *mdx* mice presented a left deviation of the electrical axis whereas none of the WT mice had a deviated axis (Figure 5a). At 12-month-old, 4 out of the 26 *mdx* mice presented a left deviation of their electrical axis (Figure 5a). Moreover, the angle of the main activation axis was highly variable (Figure 5a) though remaining in a normal range (0°–120°), showing that the stereotypical depolarization pattern is slightly affected in *mdx* hearts. To better understand the origin of the axis deviation, we analyzed the presence of fibrosis in these hearts using WGA staining which can serve as a readout of fibrosis (Emde et al., 2014). At 12 months of age, 5/7 of the *mdx* hearts showed ventricular fibrosis (Figures 5b,c). However, there was no significant correlation between fibrosis density and cardiac axis deviation (Figure 5d; r = 0.58; p = 0.06). Thus, the deviation of the main electrical axis may not result only from the presence of fibrosis in *mdx* hearts.

## Discussion

All DMD patients develop a cardiomyopathy with age associated with ventricular arrhythmias, which are the leading cause of death in these patients. In this study, we showed conduction defects worsening with age in *mdx* mice which could explain the premature death in one-quarter of *mdx* mice. ECG revealed a prolonged QRS in *mdx* mice which is associated with the progressive apparition of ventricular dyssynchrony and spontaneous premature ventricular complexes that are exacerbated by ß-adrenergic stimulation. The ventricular conduction defects and arrhythmias in *mdx* mice occur in absence of morphological anomalies in the Purkinje fibers network or connexin dysregulation but in association with I_Na_ loss and fibrosis.

In the literature, there is a strong heterogeneity in the ECG parameters recorded from *mdx* mice or in DMD patients (Perloff, 1984; Spurney, 2011; Fauconnier et al., 2010; Gavillet et al., 2006; Koenig et al., 2011). In contrast to previous studies, we found a progressive increase in PR intervals, indicative of first degree atrioventricular block. These differences may arise from the age, the sex of the animals and the conditions of ECG recordings. We performed ECG on anesthetized mice while earlier studies were performed using telemetry, and finally, measurements of ECG parameters are not as standardized in mice as in humans. Here, the follow-up of the ECG recordings revealed the progressive onset of arrhythmogenic cardiomyopathy in *mdx* mice as described in DMD patients. However, cardiac conduction defects remain mild compared to humans. We also found a constant durable QRS elongation which indicates a slow ventricular conduction. As previously shown (Megeney et al., 1999), *mdx* mice present morphological signs of cardiac hypertrophy with a larger heart and bigger cardiomyocytes which can lead to an increase of the QRS interval. Cardiac hypertrophy has been prevented in *mdx* mice using peptide-conjugated phosphorodiamidate morpholino oligomer (PPMO), however cardiac conduction has not been studied in these mice to determine whether this treatment can reduce QRS prolongation (Jearawiriyapaisarn et al., 2010).

An important feature of *mdx* mice is their susceptibility to develop spontaneous PVC which is increased by ß-adrenergic stimulation. In the vast majority of previous publications, ventricular arrhythmias arise only after isoprenaline stimulation (Gonzalez et al., 2015; Himelman et al., 2020; Lillo et al., 2019), and only one paper found PVC by telemetry in 6-month-old *mdx* mice (Fauconnier et al., 2010). The susceptibility to develop spontaneous PVC in our model may be explained by the age of the mice studied or by the conditions of ECG recording and ISO stimulation. In addition, most of our mice also carried the *Cx40-GFP* allele which could interfere with ventricular conduction. However, spontaneous and induced PVCs were also detected in *mdx* mice that do not carry the Cx40-GFP allele, suggesting that this genetic interaction may play a minor role in this arrhythmogenesis. Moreover, I_Na_ reduction induced by dystrophic-deficiency was similar from both Cx40-GFP+ and Cx40-GFP- ventricular cardiomyocytes (data not shown), suggesting that I_Na_ impairment is independent of the presence of the Cx40-GFP allele.

Our data showed an increase in the prevalence of ventricular arrhythmia with age which suggested a progressive degradation of the electrical conduction over time. To explain this phenomenon, we tested whether the Purkinje fiber network is altered as reported in human and canine DMD (Bies et al., 1992). Cx40-GFP mice were used to easily visualize the entire VCS: His bundle, bundle branches and PF network (Miquerol et al., 2004). No anomalies were observed in *mdx::Cx40-GFP* mice either in the structure or the density of the Purkinje fiber network. Thus, the VCS does not degenerate in *mdx* mice as it has been reported for human patients. Furthermore, we did not find any maturation defects in the mdx PF, which are just as numerous as in WT and express the Cntn2 marker. Although dystrophin is overexpressed in murine PFs as in humans and dogs, our data show that its absence has no effect on the formation and maintenance of the PF network in the mouse. It is well-known that PF present anatomical and histological discrepancies between mammals (Ono et al., 2009). Human and dog PFs belong to group II while those of mice belong to group III (Ono et al., 2009). Indeed, PF are thin and elongated in mice while they are larger than contractile cardiomyocytes and have a paler cytoplasm in dogs and humans. The histological difference between these species may explain the less severe damage of the PF network in dystrophic mice. Thus, our data showed that conduction defects and arrhythmias arise in *mdx* mice in absence of PF network structural defects and, in contrast to the dog, this mouse model is not suitable for translational research into cardiac conduction defects in DMD. Indeed, restored dystrophin in the heart including in PF reduced conduction defects in DMD dogs treated with micro-dystrophin (Echigoya et al., 2017).

Previous studies have suggested that conduction defects in *mdx* mice arise from a pathological mislocalisation of the gap junction Cx43 to the lateral sides of cardiomyocyte (Colussi et al., 2011; Gonzalez et al., 2015; Himelman et al., 2020; Lillo et al., 2019). Inhibiting this lateralization or blocking the activity of Cx43 hemichannels or expressing a mimicked-phosphorylated Cx43 all prevent ISO-stimulated ventricular arrhythmia and death in *mdx* mice (Gonzalez et al., 2015; Himelman et al., 2020; Lillo et al., 2019). Although, abolishing Cx43 remodeling was shown to reduce ventricular arrhythmias, conduction defects were not observed in these models (Himelman et al., 2020). These data suggest that targeting Cx43 represents a potential therapeutic strategy in the prevention of ventricular arrhythmias in human patients. Using whole-mount immunostaining, we showed that Cx43 is mainly present at the level of the IDs in contractile cardiomyocytes in both WT and *mdx* mice. In our hands, Cx43 did not show lateralization of Cx43 in contractile cardiomyocytes at the subendocardial surface in *mdx* mice. These differences may be due to the technique used or the cardiac region from which the cardiomyocytes originate. Cx43 is mainly expressed in contractile cardiomyocytes but it is also present in PF to define Purkinje-myocardium junctions (Olejnickova et al., 2021). However, in contrast to contractile cardiomyocytes, our data showed that Cx43 expression is milder and distributed all along the PF. The normally lateralized of Cx43 in PF could explain why no Cx43 mislocalisation has been observed in *mdx* mice in these cells. The impact of Cx43 on conduction defects requires further exploration to validate its significance and implications for therapy.

We observed that QRS elongation is constantly increased in *mdx* mice, however, it significatively increased in old mice. Interestingly, we found that I_Na_ loss worsens between the juvenile and the adult stage, while I_Na_ is constant in the old adults. Thus, our results suggest that I_Na_ loss in ventricular cardiomyocytes and Purkinje fibers, but not impaired ventricular conduction system development or degeneration or Cx43 dysregulation explains slowed ventricular conduction in *mdx* mice. I_Na_ reduction increases with age between juvenile to adult, however, our results show that it did not worsen at old age. However, progressive PR increase and ventricular arrhythmias that appear in *mdx* mice with age may not occur exclusively from I_Na_ decreases. Conduction defects are known to arise from the presence of fibrosis which is considered to be a non-conductive tissue (Oebel et al., 2017; Souidi et al., 2024). Progressive fibrosis has been previously described in DMD patients, dogs and mice (Marchal et al., 2021; Amedro et al., 2019; Ghaleh et al., 2023). Here, we found fibrosis in the majority of *mdx* mice and all fibrotic mice are associated with a main axis deviation. A deviation in the main cardiac axis indicates that the depolarising pattern of the ventricles is disturbed. This deviation may also indicate dyssynchrony and it is important to notice that strain defects detected by cardiac magnetic resonance are more sensitive criteria to detect cardiac dysfunction in DMD patients compared to ejection fraction (EF) (Amedro et al., 2019; Ghaleh et al., 2023). However, one limitation of this study is the limited number of mice and further experiments are necessary to determine the exact correlation between fibrosis and dyssynchrony.

Collectively, our data strongly suggest that the conduction defects in *mdx* mice are caused by the development of an arrhythmogenic cardiomyopathy associated with reduced I_Na_ and fibrosis and not by a structural remodeling of the PF network.

## Data Availability

The original contributions presented in the study are included in the article/supplementary material, further inquiries can be directed to the corresponding author.
